# Impact of COVID-19 on key populations and people living with HIV: recommendations and sociopolitical responses from the EPIC community research program in Latin America

**DOI:** 10.1186/s12889-025-22017-7

**Published:** 2025-03-12

**Authors:** Valeria Stuardo Ávila, Océane Apffel Font, Ángela León Cáceres, Pablo Radusky, Ines Aristegui, Harold Mendoza, Maria Veras, Rodrigo Pinheiro, Duvan Felipe Mesa, Miguel Ángel Barriga, Luis Gómez, Michael Reyes-Díaz, Víctor Parra, Cristian Lisboa Donoso, Lisa Kretzer, Juliana Castro Ávila, Rosemary Delabre M, Lucas Riegel, Cinta Folch Toda, Jordi Casabona, Carlos F. Cáceres, Nicolas Lorente, Daniela Rojas-Castro

**Affiliations:** 1 Universidad Andrés Bello, Santiago, Chile; 2 Escuela de Salud Publica, Universidad de Chile, Santiago, Chile; 3 Coalition PLUS, Pantin, France; 4 Heideberg University, Baden-Württemberg, Germany; 5 Universidad de Buenos Aires - Facultad de Psicología, Buenos Aires, Argentina; 6 Fundación Huésped, Buenos Aires, Argentina; 7 Insituto Para El Desarrollo Humano Bolivia, Cochabamba, Bolivia; 8 Faculdade de Ciências Médicas da Santa Casa de Sâo Paulo, São Paulo, Brazil; 9 FOAESP, São Paulo, Brazil; 10 Fundación IFARMA, Bogotá, Colombia; 11 Red SOMOS, Bogotá, Colombia; 12 CAS Guatemala, Guatemala, Guatemala; 13 Universidad Peruana Cayetano Heredia, San Martín de Porres, Peru; 14 Fundación SAVIA, Santiago, Chile; 15 Universidad de Chile, Santiago, Chile; 16 CEEISCAT, Badalona, Spain; 17 CIBERESP, Madrid, Spain; 18 SESSTIM, INSERM/IRD/Aix Marseille Université, Marseille, France; 19 Center for Interdisciplinary Studies in Sexuality, AIDS and Society, Universidad Peruana Cayetano Heredia, Lima, Peru

**Keywords:** HIV Epidemiology, COVID-19, Latin America, Public Health, Community, Health systems

## Abstract

**Background:**

Health inequality in Latin America is particularly severe for individuals living with HIV (PLHIV) and key populations, such as men who have sex with men, transgender women, people who use drugs, and sex workers. Despite regional programs aimed at reducing health inequalities, such as the Sustainable Development Goals and the Sustainable Health Agenda for the Americas 2018–2030, the COVID-19 health crisis has exposed significant shortcomings in national healthcare systems for PLHIV and key populations. The multi-country, community-based research program, EPIC, was developed by Coalition PLUS within an network of community-based organizations engaged in the response to HIV and viral hepatitis. The EPIC program aimed to study the impact of the COVID-19 health crisis on 1) key populations (KP) and/or PLHIV or hepatitis C; 2) community health workers (CHWs) and peer educators; and 3) key innovations and adaptations in HIV/HCV services. The objective of this article is to highlight main issues faced in the region during the COVID-19 health crisis in order to inform national and international policies.

**Methods:**

A general protocol and study materials were developed and included built-in flexibility to allow participating organizations to adapt the study to local needs in terms of target populations and specific areas of interest. Data were collected through surveys and/or interviews. In total 118 studies were conducted across 31 countries: 66 quantitative (*n* = 12,060 among KP or PLHIV or people living with HCV and *n* = 811 among CHWs) and 52 qualitative (*n* = 766 among KP or PLHIV or people living with HCV and *n* = 136 among CHWs).

**Discussion:**

Findings in Latin America highlight the difficulties faced by PLHIV and KP in accessing health services, as well as issues of discrimination, violence, and mental health challenges, all of which have been exacerbated by the health crisis. Additionally, the study highlights strategies implemented by community CHWs and peer educators to mitigate the negative impact of the crisis. Moreover, EPIC demonstrates the ability of community agents to generate scientific evidence that raises public awareness of the situation faced by the most vulnerable populations.

**Conclusion:**

National and international policies must recognize and support the unique capacity of CHWs and peer educators to adapt health interventions to the specific needs of communities. Policymakers are also urged to involve the community in the development of public policies aimed at reducing inequalities and improving the living conditions of vulnerable populations.

**Supplementary Information:**

The online version contains supplementary material available at 10.1186/s12889-025-22017-7.

## Background

Inequality in Latin America remains the highest among other countries globally; the wealthiest 10% of the population in Latin America has the largest share of income (37%) [1] than in any other region, globally. In comparison, the poorest 40% receive the smallest portion (13%). This situation was exacerbated due to the COVID-19 pandemic [2]. Starting in February 2020, several countries closed their borders and implemented confinement strategies [3]. This led to highly adverse economic consequences [4] and worsened health inequities in the region, especially for vulnerable populations [2, 5].

Currently, there are 3.7 million people living with HIV (PLHIV) in Latin America and 330,000 in the Caribbean, with an epidemic mostly concentrated among the key populations (KP) [6] such as men who have sex with men (MSM) (46%), transgender women (7%), and sex workers (SW) (6%) [6]. Moreover, Latin America still demonstrates considerable gaps that place it far from reaching the UNAIDS’s 95–95-95 goal: 81% of PLHIV know their status, of which only 65% are on antiretroviral treatment, and 60% of these have achieved viral suppression [6]. Additionally, approximately one third of PLHIV are diagnosed late [7]. This reflects the persistence of structural barriers to accessing HIV prevention and care services in the region, such as exposure to stigma, discrimination, and violence, as well as lack of income, employment, and educational opportunities [1].

To address this situation, it is worth noting the reference programs for policy formation in Latin America, such as the Sustainable Development Goals (SDGs) of the 2030 Agenda [8]. Goal 3.3 specifies that by 2030, we must “end the epidemics of AIDS, tuberculosis, malaria, and neglected tropical diseases and combat hepatitis.” Additionally, the Sustainable Health Agenda for the Americas 2018–2030 (ASSA2030) [7] has been implemented through the strategic plans of the Pan American Health Organization and the subregional and national health plans of the Member States. Within Goal 10 of ASSA2030, which states “reduce the burden of communicable diseases and eliminate neglected diseases,” the aim is to reduce mortality, morbidity, and stigma associated with HIV. This goal also emphasizes that achieving universal health access and universal coverage requires a comprehensive multisectoral and intersectoral approach [7].

The health crisis caused by COVID-19 has drastically reduced the application and implementation of these programs and has had unprecedented consequences on HIV care [5].

Based on the results of the international EPIC research program, this study aims to highlight Latin America’s deficiencies in the sociopolitical response to the COVID-19 crisis, especially related to vulnerable populations and PLHIV. Additionally, the study aims to propose comprehensive strategies to address the specific needs faced by these populations in the region. For example, from diverse contexts and realities, the community healthcare model implements combined strategies that integrate biomedical and community actions. Through close coordination with public institutions and direct collaboration with key populations, it aims to ensure access to health systems [9, 10].

EPIC (Community Impact Assessment Surveys for COVID-19) was an international community research program coordinated by Coalition PLUS [11], a union of non-governmental community organizations involved in HIV response in over 50 countries. The program aimed to describe the impact of the COVID-19 pandemic on PLHIV or those at risk of HIV and hepatitis C infection and on those working with these populations in community settings, known as community health workers (CHWs) and/or peer educators.

## Methods

Following the onset of the pandemic in early 2020, a mixed working group was formed by the community-based research team from Coalition PLUS, external researchers, and representatives from community organizations and key populations. Details about this group can be found elsewhere [12]. This working group actively involved partner organizations of Coalition PLUS [13], grouped by geographical, thematic, or linguistic networks. In Latin America, two networks coexist: the Americas and Caribbean Platform (PFAC), comprising 8 grassroots organizations, and the Ibero-American Network of Studies on Gay Men, Other MSM, and Transgender People (RIGHT PLUS), comprising 13 community and academic organizations (including 4 from Spain and Portugal).

The EPIC working group developed a general and adaptable protocol that foresaw the possibility of implementing qualitative and/or quantitative studies [11]. The quantitative questionnaire (see Annex 1) included a mandatory module (sociodemographic profile and general questions about the impact of COVID-19 on participants’ lives) and 11 optional thematic or population modules (e.g., access to healthcare, perceived risk of COVID-19, men who have sex with men (MSM), community health workers (CHWs), peer educators and people living with HIV (PLHIV)). Additionally, it was possible to add specific questions adapted to the local context and interest. However, two semi-structured interview guides were designed to qualitatively explore the experiences and needs of key populations and CHWs during the crisis. Informed consent to participate was obtained from all of the participants in the study.

Each organization that participated in EPIC adapted the protocol with the assistance of a template defining its own objectives, the population(s) it wanted to survey, the general methodology (qualitative and/or quantitative), and the recruitment and data collection methods. The most appropriate sampling strategy in the pandemic context in which the EPIC program was developed was to use convenience sampling. Each participating organization received technical support from the Coalition PLUS research team to reach the largest sample size based on their specific characteristics, needs and target population. Quantitative data collection was conducted using Voxco, with training provided as needed to ensure standardized procedures and centralized data management. All qualitative interviews were administered by CHWs working with the local organization in each country or region or, when necessary, by a local consultant and were recorded either in person or via teleconference in the local language, and later transcribed verbatim. In total 118 studies were conducted across 31 countries: 66 quantitative (*n* = 12,060 among KP or people living with HIV or people living with HCV and *n* = 811 among CHWs) and 52 qualitative (*n* = 766 among KP or people living with HIV or people living with HCV and *n* = 136 among CHWs). More details have been published elsewhere [11].

In Latin America, the EPIC program was implemented in seven countries: Argentina, Bolivia, Brazil, Colombia, Guatemala, Peru, and Chile (Table 1). During the implementation process, each participating organization (*n* = 9) benefited from technical support from a reference person from the research department of Coalition PLUS and from spaces for sharing experiences with other organizations throughout the region to optimize implementation and promote the development of research and communication skills. Moreover, webinars, training sessions, thematic meetings, and newsletters were organized and distributed by the Coalition PLUS community-based research department.

**Table 1:** Organizations involved in the EPIC community research program in Latin America

Country	EPIC Implementing Organizations	Other Organizations Involved in EPIC	Study Type	Target Populations (Sample Size)
**Argentina**	Fundación Huésped	Association of Transvestite, Transsexual, and Transgender Women of Argentina (ATTTA), Casa Trans, Association of Women Sex Workers of Argentina (AMMAR)	Quantitative	- Cisgender and transgender women sex workers (*n* = 173)
**Bolivia**	Instituto para el Desarrollo Humano Bolivia (IpDH)	Warmis	Qualitative	- Cisgender and transgender women sex workers (*n* = 20) - Cisgender male sex workers (*n* = 14)
**Brazil**	Núcleo de Pesquisa em Direitos Humanos e Saúde da População LGBT + (NUDHES)	Movimento de Prostitutas no Brasil, Rede Brasileira de Prostitutas (RBP), Central Única das Trabalhadoras e Trabalhadores Sexuais (CUTS), Articulação Nacional das Profissionais do Sexo (ANPROSEX), Tulipas do Cerrado, Coletivo Por Elas Empoderadas, Associação das Profissionais do Sexo da Bahia (APROSBA), Coisa de Puta, Associação das Prostitutas de Minas Gerais (APROSMIG), Associação Mulheres Guerreiras, Grupo de mulheres prostitutas do Estado do Pará (GEMPAC), Núcleo de Estudos da Prostituição (NEP), Associação Brasileira de Gays, Lésbicas, Bissexuais, Travestis, Transexuais e Intersexos (ABGLT)	Qualitative	Cisgender and transgender women sex workers (*n* = 46)
			Quantitative	Quantitative Cisgender women sex workers (*n* = 138)
	Fórum das Ongs Aids do Estado de São Paulo (FOAESP)	Jovens Unidos por Direitos Iguais e Humanos	Quantitative	People living with HIV (*n* = 107)
**Colombia**	IFARMA	Corporación Temeride, Corporación El Faro, Asociación Cimientos de Luz, Red Colombiana de Personas viviendo con VIH o con SIDA (Recolvih), Fundación Organización Acción Humanista, Fundación hogar de paso Prodihogar, Fundación amigos por la vida FUDAVIDA	Quantitative	- Drug users (*n* = 99) - Key populations, including sex workers, people living with HIV, men who have sex with men, migrant individuals (*n* = 341)
	Corporación Red Somos		Qualitative	Migrant individuals (*n* = 54)
			Quantitative	- Migrant individuals (*n* = 338) - Community health agents (*n* = 53)
**Guatemala**	Colectivo Amigos Contra el SIDA (CAS)		Quantitative	Men who have sex with men (*n* = 306)
**Peru**	Centro de Investigación Interdisciplinaria en Sexualidad, Sida y Sociedad – Universidad Peruana Cayetano Heredia (CIISSS-UPCH)		Qualitative	- Transgender women (*n* = 9) - Men who have sex with men (*n* = 10)
			Quantitative	Men who have sex with men (*n* = 476)
**Chile**	Instituto de Salud Pública Universidad Andrés Bello/Escuela de Salud Pública, Universidad de Chile	Fundación SAVIA	Quantitative	People living with HIV (*n* = 249)

All data presented in this article come from descriptive analyses for the quantitative studies (frequencies), and thematic analyses for the qualitative studies.

## Results

### Access to healthcare during the health crisis

Corroborating other recently published studies [4, 14–17], the results of the EPIC program in Latin America highlight the following key issues related to healthcare access during the health crisis:

1. Difficulties to access to healthcare in general and for PLWHIV
2. Arduousness to get antiretrovirals for those living with HIV
3. Poor access to prevention tools for key populations in different countries during the crisis

According to quantitative data collected in Colombia, 20% of drug users perceived a decrease in access to prevention tools and/or services, 14% experienced a decrease in access to treatments, and 6% saw a decrease in access to harm reduction tools and/or services related to drug use. The closure of healthcare services was the main barrier to accessing prevention, diagnosis, and treatment for this population. Conversely, for migrants in this country, the lack of valid identification documents or visas or a COVID-19 vaccination card posed the most significantbarriers to receiving care at health centers.

In Peru, during the lockdown, dispensing medication to PLHIV was prioritized, which caused the neglect of prevention services. Data from Peru showed that 57% of MSM who were using daily or on-demand pre-exposure prophylaxis before the health crisis stopped taking it during the lockdown, and 45% of them resumed taking it when the lockdown ended.

Difficulty in accessing healthcare services was also evident among sex workers in the qualitative study in Bolivia. In this regard, the closure of HIV testing centers and of the administration renewing the health card required to engage in sex work was one of the barriers to accessing sexual health services during the pandemic. The lack of money to pay for consultations or tests in private clinics and the fear of contracting COVID-19 were other identified barriers. Community organizations played a key role in compensating for the closure of these services. One participant summarized it as follows: “*Throughout this COVID-19 pandemic, I haven’t visited any hospital or anything like that because of the fear as well. But yes, at the Instituto para el Desarrollo humano IpDH, I have been able to call and consult”* (cisgender woman, 32, Bolivia).

According to the results of another EPIC study in Colombia, healthcare for migrants during the COVID-19 pandemic was scarce, as reported by the 53 interviewed migrants following qualitative guidelines, who stated that without a health card or documents required by the government, they did not receive care in healthcare centers.

Regarding access to antiretroviral treatment, in Chile, 23% of the surveyed PLHIV reported having experienced difficulties accessing treatment, among which difficulties in traveling to the service (82%) and difficulties in renewing medical prescriptions (46%) stood out. Of the participants, 57% had reduced follow-ups with their doctors, 11% had difficulties with the treatment because they were confined with people unaware of their seropositivity, and approximately 20% reported worse adherence to antiretroviral treatment since the beginning of the health crisis.

EPIC data also highlight the adaptability of certain healthcare services to address these access issues during times of crisis. In Chile, for example, the most reported adaptative solutions in the survey among PLHIV were (multiple answer question): dispensing antiretroviral treatments for an extended period (39%), reducing the frequency of HIV follow-up appointments (25%), and telemedicine consultations (33%). In Bolivia, telemedicine was reported in the qualitative study as a facilitator for receiving care or addressing urgent physical and mental health issues in male and female sex workers. This healthcare strategy was mainly carried out with known healthcare professionals or agents, without a prior appointment, and as a medical follow-up.

### Discrimination, violence, and stigma

PLHIV and key populations continue to be discriminated against, stigmatized, and victims of various forms of violence today [9, 18–22]. Since the onset of the health crisis caused by COVID-19, various studies have shown that these problems have intensified [23–25], and the data from EPIC also support this trend.

In Peru, transgender women interviewed in the EPIC program experienced double economic discrimination during the pandemic. In addition to being excluded from formal and stable jobs as usual, they lost their sources of income and were not included in the list of vulnerable populations eligible to receive support packages from the government (economic or food). Additionally, they were victims of transphobia by the police within the context of mobility restrictions: *“Sometimes people said it was something authoritarian, the lockdown. The most talked-about issue was on which day males had to go out and on which day females had to go out, so there was a lot of bullying and discrimination. […] a police officer mocked a girl by making her do frog jumps; I want to be a man, I want to be a man. […] Discrimination against our community was very clear, especially against our transgender community” *(transgender woman, 23, Peru).

In Argentina, during the pandemic, there was an increase in police violence, exacerbated by the lack of a legal framework guaranteeing the rights of sex workers and regulating their activity. According to EPIC data on cisgender and transgender sex workers, 45% experienced violence (physical, verbal, psychological, or sexual) by security forces during that period, and 77% reported that police violence had become more frequent since the onset of the pandemic. Furthermore, 34% reported an increase in the frequency of experiences of discrimination by healthcare workers.

Access to the COVID-19 vaccine was also a source of stigmatization for certain populations. In Chile, EPIC results showed that 60% of PLHIV had to disclose their serological status in some way to access it. The majority of people (67%) considered that having to disclose their serological status had negative consequences for them, citingin stigma (21%), discrimination (12%), and psychological violence (11%).

### Other consequences of the pandemic

Beyond the issues of access to healthcare services and the discrimination, stigma, and violence experienced by key populations, EPIC data also highlight the economic consequences of the pandemic and the impact on the quality of life and mental health of these populations, as noted in previously published literature [15, 24, 26–29].

In Guatemala, EPIC showed that 27% of surveyed MSM reported that their sexual quality of life had deteriorated slightly or greatly since the onset of the COVID-19 pandemic. Additionally, 46% of respondents reported that their economic situation had deteriorated slightly or greatly since the beginning of the pandemic, and 19% reported that their general quality of life had deteriorated slightly or greatly since the onset of the pandemic. The impact on the key population’s mental health was significant: 32% had mild anxiety symptoms and 28% had moderate to high anxiety (generalized anxiety disorder scale [30]).

In Bolivia, sex workers interviewed using qualitative guidelines saw their economic needs exacerbated during the health crisis. The decrease in the number of clients and/or rates caused by the closure of public places and lockdowns even created difficulties related to access to food:

*“(...) well, no, there was no work [during the quarantine], there wasn’t much, and all the girls were desperate, I think there were more girls than clients (...). Up to this day, there aren’t many clients, even with the raids [raids conducted by the police] now, it’s worse; clients are scared and don’t want to go to the venues. It’s all empty.*

*(...) And well, we’ve been economically affected (...), and you know debt and food don’t wait (...). They even kicked me out of the house. Now, I’m in another one (...) because I couldn’t pay the rent; we couldn’t even get to the market.”*

The same situation was found in Brazil, where 81% of female sex workers reported loss of clients and decrease in income, with sex work being the only source of income for 68% of them.

## Discussion

### Encouraging community participation and alignment between health services and needs

From community experience and scientific evidence, community organizations have evinced that addressing the HIV epidemic requires both biomedical and social strategies to end inequalities and achieve HIV elimination by 2030 [10]. The results obtained within the framework of the EPIC community research program, primarily led by community organizations themselves, highlight the numerous negative consequences of the health crisis on vulnerable populations and, consequently, the shortcomings of national health systems in responding to the needs of these populations.

In EPIC data, many participants reported that assistance received during the health crisis, including social, health, and economic, came from the community organizations that reacted swiftly. From diverse realities and contexts, the community model approach proposes combined strategies involving biomedical and community actions, establishing a high level of coordination with public institutions to ensure access to health systems through organizing actions and close collaborations with key populations [9, 10]. Thus, the complementarity of health systems is emphasized, with community organizations acting as the bridge between key populations and the national healthcare system, while highlighting the strategy of peers and community health workers and peer educators in their broad diversity. From the perspective that ensures human rights, it is also imperative to combat stigmatization and discrimination in the health sector, as well as among its professionals, especially related to HIV and key populations. In that sense, community-based organizations are well placed to train health professionals in order to sensibilize them to these issues.

The United Nations General Assembly’s Political Declaration on HIV reinforces this idea by affirming that community-led organizations should be responsible for at least “30% of testing and treatment services. This includes ‘HIV screening tests, connections to service providers and education related to treatment and therapeutic regimen adherence.’” It adds that they should also manage “80% of HIV prevention services for populations at high risk of infection, including women” by 2025.

As illustrated by the EPIC results discussed above, public policies should emphasize guaranteeing and facilitating universal access to health services and HIV and sexually transmitted infections prevention methods, including for migrants. Moreover, some of these services should be provided at the community level as part of the effort to reduce inequality and improve living and health conditions for vulnerable populations. Therefore, it is vital to strengthen community services closer to key populations and establish an emergency plan to maintain a minimum health and prevention service during crises.

Limitations.

The pandemic context may have limited some straightforward questions, such as difficulties in obtaining ethical committee approval, as these committees halted their activities. Additionally, challenges in accessing the field arose as waves of COVID-19 led to new lockdowns, resulting in data collection being extended over time. This had some effects on the analysis, even though adjustments were made, such as incorporating changes in types of lockdowns and public health regulations to reduce biases. The autonomy given to each community-based organization to choose their scope and methods made it impossible to conduct a comprehensive global analysis of the quantitative data. Regarding the qualitative aspect, the fact that some of the CHWs conducting the interviews were doing so for the first time impacted the quality of the interviews, and the data may not have allowed for an in-depth analysis.

## Conclusion

As a public health problem, the COVID-19 pandemic has challenged decades of progress toward achieving the SDGs by disrupting essential health services, halting progress toward universal health coverage, and hampering progress toward eliminating HIV/AIDS. Based on the results of the EPIC program, it is clear that national health systems still require work and effort to achieve the SDGs. In the regional agenda, addressing access to healthcare in vulnerable populations, defending human rights, combating stigma and discrimination against PLHIV and key populations, and mitigating the negative health, social, and economic consequences left by the social-sanitary crisis must be prioritized. It is indispensable to increase the participation of civil society and communities in policy formulation and in the evaluation process related to health in all policies in order to reduce health inequities [7] ( given the comparative advantage of community-led HIV responses [31]). Furthermore, to adopt policies that allow for the sustainable financing of integrated people-centered community responses through various public financing mechanisms [32]. Finally, given the experiences and capacities at the community level related to addressing health crisis situations, strengthening communication and social mobilization capacities in the context of epidemic outbreaks is key to the success of the response [33, 34]. The incorporation of socio-cultural aspects, as well as the challenges or facilitators of implementing strategies in the most vulnerable populations, can quickly contribute to the response and actions of governments in facing such health emergencies.

To achieve this, community systems and models are essential. Community response during the crisis through concrete actions (e.g., community distribution of antiretrovirals, social and psychological support to vulnerable populations, development of telemedicine, and so on) along with the involvement of community health workers and peer educators in community research projects like EPIC, demonstrate that they can efficiently contribute to improving population health in times of crisis. Those responsible for policy formation and implementation must promote and institutionalize community participation both at the level of healthcare services and in scientific research.

## Supplementary Information

Supplementary Material 1
