# Supplementary material for: Impact of COVID-19 on key populations and people living with HIV: recommendations and sociopolitical responses from the EPIC community research program in Latin America
Source: BMC Public Health. 2025 Mar 12;25:971. doi: 10.1186/s12889-025-22017-7 (PMC11898992; doi:10.1186/s12889-025-22017-7)
Supplement: Supplementary file 1 — Supplementary Material 1 [file 12889_2025_22017_MOESM1_ESM.docx]

| **EPIC: A multi-country study to assess the impact of Covid-19 among populations vulnerable to, or living with, HIV and/or HCV, and among people who work with these populations in the community setting** |
| --- |

# Questionnaire for key populations

***Version EN 1.0 : 30 December 2020***

***Translation of the version 2.0 dated 16 décembre 2020***

**The questionnaire is organised in 12 modules :**

- B1 : Core module
- B2 : Sexual activity and prevention strategies for HIV and other STIs
- B3 : Experience and application of lockdown and/or prevention measures
- B4 : Socio-economic needs and responses in relation to the COVID-19 health crisis
- B5 : Representation and Perception of COVID-19 risk
- B6 : Psychological well-being and resilience
- B7 : Access to healthcare
- B8 : People who use drugs (PWUD)
- B9 : People living with HCV (PLHCV)
- B10 : Pre-exposure prophylaxis (PrEP)
- B11 : People living with HIV (PLHIV)
- B12 : Sex workers (SW)

The core module is the only compulsory module for all questionnaires. Only one response is allowed for the questions unless “*multiple answers possible*” is mentioned.

*[****italic****]:* filters (for example [IF FILTER: “Yes” to Q7] means that the question opens if the answer “Yes” has been selected in question 7),

[**underlined**]: technical elements (eg exclusive questions in a multiple choice).

→ It is not necessary to translate these elements.

| B1 : “Core module” |
| --- |

This section includes some general questions that will help us get to know you better. We remind you that this questionnaire is completely anonymous.

1. **How old are you? ______ years [exclusion: <18 years]**
2. **Currently, you define yourself as***: (Multiple answers possible)*

- A woman
- A man
- A transgender woman
- A transgender man
- Other, specify: ____________________
- I do not wish to answer

1. **What is your highest validated education level?**

- I never went to school
- Primary (or elementary) level
- Secondary level
- Third level (or Higher education), including: university and college
- Other, specify: _____________________
- I do not wish to answer

1. **What is your country of residence? _________________________ (country list)**
2. **What is your country of birth? _________________________ (country list)**
3. ***[ASK THIS QUESTION ONLY IF: the country of birth (Q5) is different from the country of residence (Q4)]*****

**What is your administrative situation in the country where you currently live?**

- Regularized (visa, residence permit, etc.)
- Not regularized (no papers, expired visa / expired residence permit, etc.)
- Awaiting a visa or papers for regularization
- I do not wish to answer

1. **Currently, you are living:**

- In an urban area / in a large town or city
- In a semi-urban area / in a small or medium-sized town
- In a rural area / in a village

**7.2 Currently, you are living:**

❏ In a housing that I own

❏ In rented housing

❏ In housing where I am staying for free

❏ In a squat

❏ On the street / Homeless

❏ I do not wish to answer

1. **Among the following groups, tick the ones you identify with *(Tick at least one box. You can tick more than one box if necessary)***

- Men who have sex with men
- Gays and lesbians
- Transgender people
- Sex workers
- People who inject drugs
- People who use non-injecting drugs (smoking, inhalation, etc.)
- People living with HIV
- People living with HCV
- People living with another chronic disease (not HIV or HCV)
- Migrants
- I do not identify with any of these groups **[exclusive choice]**
- I do not wish to answer **[exclusive choice]**

1. **Since the start of the Covid-19 health crisis, has your financial situation changed compared with the period before the epidemic?**

- Yes, I am more financially comfortable
- No, my financial situation has not changed
- Yes, my financial situation has deteriorated a little
- Yes, my financial situation has deteriorated a lot

1. **Have you already sought help from the [*Coalition PLUS local structure’s name] association?***

- No, never
- No, but I would like to
- Yes, before the Covid-19 health crisis
- Yes, before and during the Covid-19 health crisis
- Yes, following the onset of the Covid-19 health crisis

1. **Compared with before the Covid-19 crisis, how would you evaluate … ?**

|  | Much better | Somewhat better | More or less the same | Somewhat worse | Much worse | I do not wish to reply |
| --- | --- | --- | --- | --- | --- | --- |
| Your current quality of life | □ | □ | □ | □ | □ | □ |
| The current **quality of your sex life**  (with partner (s) who are not commercial/ transactional sex clients) | □ | □ | □ | □ | □ | □ |

1. **Since the start of the Covid-19 health crisis, with regard to your sex life with partner(s) who are not commercial/transactional sex clients:** *(Multiple answers possible)*

- I have had sex
- I have suffered from a lack of sexual activity
- I have made appointments for "after"
- I have reduced sexual encounters with new partners
- I have increased sexual encounters with new partners
- I have had more sex with the partner who lives with me
- I have had less sex with the partner who lives with me
- I have had more sexual activities / chats / videos via messaging
- During sex, I have reduced my use of prevention methods (condoms, PrEP, etc.)
- None of the above **[exclusive choice]**

1. **How would you evaluate the impact of the Covid-19 health crisis on:**

|  | It is very negative | There is more negative than positive | Nothing much has changed with respect to before | There is more positive than negative | It is very positive | I do not wish to respond | Not applicable |
| --- | --- | --- | --- | --- | --- | --- | --- |
| Your personal life in general | □ | □ | □ | □ | □ | □ | □ |
| Your professional life in general | □ | □ | □ | □ | □ | □ | □ |

1. **Do you have the intention to get vaccinated when a vaccine which is effective and recommended by your country's health authorities, becomes available?**

- Yes, definitely
- Yes, probably
- Maybe
- No, probably not
- No, definitely not

1. ***[FILTER Show if “No” or “maybe” to Q14]* Among these different obstacles to vaccination, which are the most important for you?** *(several answers possible, maximum 3)***:**

- I do not think I will have the financial means to get vaccinated
- It will be difficult for me to access the vaccination facilities
- I am afraid of being discriminated against by going to get vaccinated
- I do not trust the Covid-19 vaccine
- I have concerns about the risks and side effects of the Covid-19 vaccine
- I do not believe in vaccination in general
- I have already had or think I have had Covid-19
- I consider the risk of catching Covid-19 to be low
- I would rather catch Covid-19 than get vaccinated
- I have a pre-existing health problem
- Other, give details : _____

1. ***[FILTER Show if “Yes” or “Maybe” to Q14]* Among the following places, where would you prefer to get vaccinated?** *(several answers possible, maximum 3)*

- At my general practitioner
- At the hospital / dispensary / healthcare center
- At the pharmacy
- In a public place (public vaccination campaigns)
- At home
- At my workplace / school
- In an association / community health center
- During a preventive action (outside: in the street / places of conviviality / outreach) *(to be adapted)*
- Other, give details :
- I do not know **[exclusive]**

| B2 : Sexual activity and prevention strategies for HIV and other STIs |
| --- |

This section concerns your sex life. Although some questions may seem very personal, remember that the questionnaire is anonymous. Please note, for people who have commercial or transactional sex, this section only concerns your non-commercial/non-transactional sexual partners (i.e., it excludes sex work).

1. **Since the start of the Covid-19 health crisis, how has the number of your sexual partners changed compared to before the crisis?**

- It has greatly decreased
- It has decreased
- It has neither decreased nor increased
- It has increased
- It has greatly increased
- Not applicable

1. **Since the start of the Covid-19 health crisis, how has the frequency with which you have sex changed compared to before the crisis?**

- It has greatly decreased
- It has decreased
- It has neither decreased nor increased
- It has increased
- It has greatly increased
- Not applicable

1. **When did you last have sex?**

- Less than 48 hours ago
- Between 2 and 7 days ago
- Between 8 and 14 days ago
- A long(er) time ago
- I have never had sex

1. **Since the start of the Covid-19 health crisis, from a HIV risk perspective, would you say that your sexual activity has been?**

- Much less risky than before
- Less risky than before
- Neither more nor less risky than before
- More risky than usual
- Much more risky than before
- Not applicable

1. **Since the start of the Covid-19 health crisis, from an STI risk perspective, would you say your sexual activity has been?**

- Much less risky than before
- Less risky than before
- Neither more nor less risky than before
- More risky than usual
- Much more risky than before
- Not applicable

1. **Compared with before the Covid-19 health crisis, how has the frequency with which you use the following risk reduction methods changed?**

|  | **More frequent use** | **Similar frequency to before the health crisis (no change)** | **Less frequent use** | **I have never used this risk reduction tool/ method** |
| --- | --- | --- | --- | --- |
| a. Condoms | □ | □ | □ | □ |
| b. Pre-exposure prophylaxis (PrEP)  This is a medical treatment taken by HIV-negative people which protects them from HIV infection | □ | □ | □ | □ |
| c. Post-exposure prophylaxis (PEP) or emergency treatment  This is a medical treatment taken by HIV-negative people after a high-risk exposure to infection | □ | □ | □ | □ |
| d. Antiretroviral therapy for treatment as prevention (TasP)  This is a medical treatment taken by HIV-positive people which protects their partners from HIV infection if taken correctly | □ | □ | □ | □ |
| e. Adaptation of sexual practices according to your serological status and that of your partners (also known as Seroadaptation) | □ | □ | □ | □ |
| f. Choosing partners according to their serological status (also known as Serosorting) | □ | □ | □ | □ |

1. **Compared to before the Covid-19 health crisis, how has the frequency with which you participate in the following practices changed?**

|  | **More frequent use** | **Same frequency as before** | **Less frequent use** | **Not applicable** | **I do not wish to reply** |
| --- | --- | --- | --- | --- | --- |
| 1. Use of drugs in a sexual context (also known as Chemsex) | □ | □ | □ | □ | □ |
| 1. Injection drug use in a sexual context (called Slamming) | □ | □ | □ | □ | □ |

| B3 : Experience with and application of lockdown and/or prevention measures |
| --- |

This section concerns your experience of the Covid-19 health crisis, and your use or application or not of prevention measures, as well as lockdown conditions.

1. **During the past 7 days, which of the following protective measures against COVID-19 have you taken or used?**

*Please first tick all the boxes for the measures you have adopted (“Yes”) or not adopted (“No).*

*Then looking at the list of measures you have used, tick those (maximum 3) which have been the most restrictive for you. [ You can also choose to tick no boxes]*.

| **Prevention measures** | **Used** | **Please tick the 3 measures that were most restrictive for you [Tick a minimum of one box – possibility to select none, maximum of 3 choices]** |
| --- | --- | --- |
| a. Wearing a mask in public | - Yes - No |  |
| b. Using hydroalcoholic hand sanitizing gel | - Yes - No |  |
| c. Stocking up on food for fear of shortages | - Yes - No |  |
| d. Stocking up on food to limit going outside | - Yes - No |  |
| e. Regular handwashing | - Yes - No |  |
| f. Avoiding public transport | - Yes - No |  |
| g. Keeping your children (if any) at home as much as possible | - Yes - No |  |
| h. Staying at home as much as possible | - Yes - No |  |
| i. Working from home and not going to the workplace | - Yes - No |  |
| j. Limiting all forms of social interaction (meeting family or friends, drinks/happy hour, etc.) | - Yes - No |  |
| k. Keeping a distance of at least one metre from other people in shops, markets and other public places | - Yes - No |  |
| l. Coughing and sneezing in your elbow | - Yes - No |  |
| m. Using disposable tissues and throwing them away after one use | - Yes - No |  |
| n. Greeting people without shaking hands and not hugging people who do not live with you | - Yes - No |  |

1. **Are you currently in lockdown (whether voluntary or imposed)?**

- Yes
- No *(go to question 4)*

1. ***[ASK THIS QUESTION ONLY IF “Yes” to Q2*] If you replied “yes” to question 2, what date did you start lockdown?**

**___/___/___ (DD/MM/YYYY)**

1. ***[ASK THIS QUESTION ONLY IF “No” to Q2]* If you replied “no” to question 2, have you experienced one or more periods of lockdown due to the Covid-19 health crisis?**

- Yes, and the last lockdown finished on the **___/___/___ (DD /MM/YYYY)**
- No (please go directly to the next section of the questionnaire)  **[If “No” to question 2 AND to question 4 → end of module]**

1. ***[ASK THIS QUESTION ONLY IF: “Yes” to Q2 OR “Yes” to Q4]***

**During the last lockdown, are you / were you living in..?**

- A house
- An apartment
- Collective housing (hostel, half-way house, other)
- Unstable housing (caravan, squat, car, tent, etc.)

1. ***[ASK THIS QUESTION ONLY IF: “Yes” to Q2 OR “Yes” to Q4*] In the place where you are/were during the last lockdown, do/did you have access to the following goods/services?** *(Multiple answers possible)*

- Basic services (drinking water, electricity, sanitation, gas)
- Internet connection
- Computer or tablet
- Smartphone
- Natural light (windows)
- None of the above **[exclusive choice]**

1. ***[ASK THIS QUESTION ONLY IF: “Yes” to Q2 OR “Yes” to Q4]***

**How would you evaluate your housing conditions during the last lockdown?**

- Very satisfactory
- Quite satisfactory
- Neither satisfactory nor unsatisfactory
- Unsatisfactory
- Not at all satisfactory

1. ***[ASK THIS QUESTION ONLY IF: “Yes” to Q2 OR “Yes” to Q4] Are/were you* in lockdown with other people?**

- Yes, with another person
- Yes, with 2-4 other people
- Yes, with more than 4 other people
- No, alone

1. ***[ASK THIS QUESTION ONLY IF: “Yes” to Q2 OR “Yes” to Q4 AND “No, alone” to Q8]* Are/were you in lockdown with….?** *(Multiple answers possible)*

- One or more children
- One or more people over 65 years old
- One or more people with chronic pathologies associated with a risk of Covid-19 (e.g., diabetes, hypertension, obesity)
- None of the above **[exclusive choice]**

1. ***[ASK THIS QUESTION ONLY IF: “Yes” to Q2 OR “Yes” to Q4]* Over the period of the last lockdown, have you/did you respect all government measures:**

- Yes, absolutely
- Yes, most of them
- Sometimes yes, sometimes no
- No, not really
- No, not at all
- I do not wish to answer

1. ***[ASK THIS QUESTION ONLY IF: “Yes, most of them” OR “Sometimes yes, sometimes no” OR “No, not really”, OR “No, not at all”, to Q10****]*

**If you did not always respect the lockdown, what were the reasons?** *(Multiple answers possible)*

- I needed to get psychoactive products (OST, drugs, etc.)
- I needed to have sex with sexual partners outside of home
- I needed to see my close family/friends
- I could not stand not being alone anymore
- I could not stand being alone anymore
- I do not have stable housing
- I do not think the lockdown is really useful
- I think that the lockdown is an attack on freedoms
- Other, specify: __________________

1. ***[ASK THIS QUESTION ONLY IF: “Yes” to Q2 OR “Yes” to Q4 AND “No, alone” to Q8]*** **Have you felt an impact of the lockdown on the frequency of arguments or family tensions in your household?**

- Yes
- No

1. ***[ASK THIS QUESTION ONLY IF: “Yes” to Q12]* Have there been more serious arguments or a climate of violence in your household?**

- Yes, much more often
- Yes, more often
- Yes, sometimes
- No, not really
- No, never

1. ***[ASK THIS QUESTION ONLY IF “Yes, much more often” OR “Yes, more often” OR “Yes, sometimes”, to Q13]* Has there been one or more people hit or physically injured in your household?**

- Yes
- No
- I do not wish to reply

1. ***[ASK THIS QUESTION ONLY if “Yes, much more often” OR “Yes, more often” OR “Yes, sometimes”, to Q13]* Have you been forced to have sex against your will, because of verbal, physical or any other kind of pressure in your household?**

- Yes
- No
- I do not wish to reply

1. ***[ASK THIS QUESTION ONLY IF: “Yes” to Q2 OR “Yes” to Q4] In* general, how would you evaluate the impact of lockdown on your personal life?**

- It is very negative
- It is more negative than positive
- Nothing much has changed with respect to my life before
- It is more positive than negative
- It very positive

1. ***[ASK THIS QUESTION ONLY: “Yes” to Q2 OR “Yes” to Q4]* In general, how would you evaluate the impact of the lockdown on your work in general?**

- It is very negative
- It is more negative than positive
- Nothing much has changed with respect to my life before
- It is more positive than negative
- It is very positive
- Not applicable

| B4: Socioeconomic needs and responses during the Covid-19 health crisis |
| --- |

This section discusses the socioeconomic needs you have experienced and the response to those needs.

1. **Before the Covid-19 health crisis, did you need any of the following types of aid?**

|  | **Yes, but I did not receive any** | **Yes, and I received it** | **No, I did not need any** |
| --- | --- | --- | --- |
| a. Food aid | □ | □ | □ |
| b. Financial aid | □ | □ | □ |
| c. Another type of material aid or a helping hand | □ | □ | □ |
| d. Information-based support: advice, discussions, information | □ | □ | □ |
| e. Moral, emotional or psychological support | □ | □ | □ |

1. **Since the start of the Covid-19 health crisis, have you needed any of the following types of aid***? (Multiple answers possible)*

|  | **Yes, but I have not received any**  **[exclusive choice]** | **Yes, and I have received it from my loved ones**  (family, friends, neighbours, peers, etc.) | **Yes, and I have received it from an association**  (charitable, social, religious or community-based, etc.) | **Yes, and I have received it by other means** | **No, I have not needed any**  **[exclusive choice]** |
| --- | --- | --- | --- | --- | --- |
| a. Food aid | □ | □ | □ | □ | □ |
| b. Financial aid | □ | □ | □ | □ | □ |
| c. Another type of material aide or a helping hand | □ | □ | □ | □ | □ |
| d. Information-based support: advice, discussions, information | □ | □ | □ | □ | □ |
| e. Moral, emotional or psychological support | □ | □ | □ | □ | □ |

1. **Since the start of the Covid-19 health crisis, in your daily life, have you had any difficulties concerning:**

|  | **More often than before** | **Same as before (no change)** | **Less often than before** | **I have never had this difficulty** |
| --- | --- | --- | --- | --- |
| a. Food | □ | □ | □ | □ |
| b. Accommodation | □ | □ | □ | □ |
| c. Financial resources | □ | □ | □ | □ |
| d. Childcare | □ | □ | □ | □ |
| e. Physical safety | □ | □ | □ | □ |
| f. General health | □ | □ | □ | □ |
| g. Social support (friends, family) | □ | □ | □ | □ |
| h. Access to Hepatitis C care | □ | □ | □ | □ |
| i. Access to HIV care | □ | □ | □ | □ |
| j. Other chronic diseases | □ | □ | □ | □ |
| k.Washing hands/access to sanitation services | □ | □ | □ | □ |

1. **In your opinion, the economic impact of the Covid-19 health crisis in your country is…**

- Not at all worrying
- Not really worrying
- Neither worrying *nor* not worrying
- Somewhat worrying
- Very worrying

1. **With regard to the next few weeks, to what extent do you think you will need the following types of aid?**

|  | **Extremely likely** | **Likely** | **Neither likely nor unlikely** | **Unlikely** | **Extremely unlikely** |
| --- | --- | --- | --- | --- | --- |
| a. Financial aid | □ | □ | □ | □ | □ |
| b. Food aid | □ | □ | □ | □ | □ |
| c. Another type of material help or a helping hand | □ | □ | □ | □ | □ |
| d. Information-based support: advice, discussions, information | □ | □ | □ | □ | □ |
| e. Moral, emotional or psychological support | □ | □ | □ | □ | □ |

| **B5 : Representation and Perception of COVID-19 risk** |
| --- |

The aim of this section is to assess your risk perception of infection by the virus responsible for the Covid-19 disease, and the extent to which you feel you would be able to protect yourself and cope if you were infected.

1. **Here are several claims about the virus responsible for Covid-19. Please indicate for each of them whether, in your opinion, they are true or false.**

|  | **True** | **False** | **I do not know** |
| --- | --- | --- | --- |
| 1. The virus can only be transmitted by people who have symptoms such as a fever and a cough |  |  |  |
| 1. The virus can be transmitted by people who do not have symptoms |  |  |  |

1. **This question has been deleted – keep this line to respect the numbering of the questions.**
2. **How would you evaluate the severity of Covid-19 on your health if you contracted it?**

- Very serious
- Somewhat serious
- Neither serious nor not serious
- Not really serious
- Not at all serious

1. **Personally, do you feel that you are capable of respecting or using protective measures to avoid catching the virus responsible for Covid-19?**

- Yes, absolutely
- Somewhat, yes
- Neither capable nor incapable
- Not really, no
- No, not at all

1. **Have you had any symptoms or signs of illness since February that made you think that you had Covid-19?**

- Yes, but I did not do a test
- Yes, I had a test and the result was positive
- Yes, I had a test and the result was negative
- No
- I do not know
- I do not wish to reply

1. ***[ASK THIS QUESTION ONLY IF: “Yes, but…” OR “Yes…positive”  OR “Yes…negative” to Q5]*** **Following these symptoms or signs of the disease, what did you do?** *(Multiple answers possible)*

- I called a doctor
- I went to a doctor for a consultation
- I called for emergency help
- I went to the emergency department
- I was hospitalized
- I self-isolated
- I did none of the above **[exclusive choice]**

1. **How would you evaluate your risk of contracting Covid-19 in the next few months?** *(Multiple answers possible)*

- Very high
- High
- Neither high nor low
- Low
- Very low
- I do not know

1. **Compared to other people in your country’s population - of the same sex, of the same age, and in the same state of health as you, how would you evaluate your risk of getting infected with this new virus?**

- Much higher
- Somewhat higher
- More or less the same
- Somewhat lower
- Much lower

1. **Among the following infectious risks, please tick those that you are most worried about** *(Multiple answers possible- maximum 3 answers)*

- HIV
- Hepatitis C virus (HCV)
- Hepatitis B virus (HBV)
- Tuberculosis
- Malaria
- Covid-19
- Ebola
- Dengue
- Other, specify: __________
- None **[exclusive choice]**

1. **With regard to the weeks and months to come** *(Multiple answers possible- maximum 3 answers)* **[minimum 1 , maximum 3 selected choices] :**

- I fear a rebound of the epidemic
- I will be happy to return to a “normal” life
- I fear a new kind of social interaction that does not suit me
- I fear a major economic crisis for society
- I fear the economic consequences of the crisis for me, my family and friends.
- I do not trust the government's ability to handle this crisis
- I have confidence in our ability to act as a society
- I do not trust others to respect protection measures
- I fear that people with Covid-19 (whether real or suspected) will be mistrusted and discriminated against.
- I think the epidemic will be brought under control
- I fear the loss of individual freedom linked to tracing (sharing of personal data)
- I fear negative changes in working conditions
- I will be happy to see my work colleagues again
- I think there will be positive changes in working conditions
- I trust others to respect protection measures
- I will be happy to see my loved ones again
- None of the above **[exclusive choice]**

| B6: Psychological well-being and resilience |
| --- |

This section aims to assess the impact of the Covid-19 health crisis on your psychological well-being, as well as your ability to overcome crises (resilience).

1. **Over the last 2 weeks, how often have you been bothered by any of the following problems?**

For each item, tick the box () that best corresponds to your situation:

|  | **Not at all** | **Several days** | **More than half the days** | **Nearly every day** | **Currently, is it more often than before the Covid-19 crisis?** |
| --- | --- | --- | --- | --- | --- |
| a. Little interest or pleasure in doing things | □ | □ | □ | □ | □ Yes  □ No |
| b. Feeling down, depressed or hopeless | □ | □ | □ | □ | □ Yes  □ No |
| c. Trouble falling or staying asleep, or sleeping too much | □ | □ | □ | □ | □ Yes  □ No |
| d. Feeling tired or having little energy | □ | □ | □ | □ | □ Yes  □ No |
| e. Poor appetite or overeating | □ | □ | □ | □ | □ Yes  □ No |
| f. Feeling bad about yourself—or that you are a failure or have let yourself or your family down | □ | □ | □ | □ | □ Yes  □ No |
| g. Trouble concentrating on things, such as reading the newspaper or watching television | □ | □ | □ | □ | □ Yes  □ No |
| h. Moving or speaking so slowly that other people could have noticed? Or the opposite—being so fidgety or restless that you have been moving around a lot more than usual | □ | □ | □ | □ | □ Yes  □ No |
| i. Thoughts that you would be better off dead or of hurting yourself in some way | □ | □ | □ | □ | □ Yes  □ No |

1. ***[ASK THIS QUESTION ONLY IF: at least one of the problems from B6 Q1 have been reported (not equal to “not at all”)]*** **If you checked off any problems, how difficult have these problems made it for you to do your work, take care of things at home, or get along with other people?**

- Not difficult at all
- Somewhat difficult
- Neither difficult *nor* not difficult
- Very difficult
- Extremely difficult
- Not applicable

1. **During the past 2 weeks, how often have you been bothered by the following problems?**

For each item, tick the box () that best corresponds to your situation:

|  | **Not at all** | **Several days** | **More than half the days** | **Nearly every day** | **Currently, is it more often than before the Covid-10 crisis?** |
| --- | --- | --- | --- | --- | --- |
| a. Feeling nervous, anxious or on edge | □ | □ | □ | □ | □ Yes  □ No |
| b. Not being able to stop or control worrying | □ | □ | □ | □ | □ Yes  □ No |
| c. Worrying too much about different things | □ | □ | □ | □ | □ Yes  □ No |
| d. Trouble relaxing | □ | □ | □ | □ | □ Yes  □ No |
| e. Being so restless that it is hard sit still | □ | □ | □ | □ | □ Yes  □ No |
| f. Becoming easily annoyed or irritable | □ | □ | □ | □ | □ Yes  □ No |
| g. Feeling afraid as if something terrible could happen | □ | □ | □ | □ | □ Yes  □ No |

1. **Since the start of the Covid-19 health crisis, you sleep:**

- Much better than before
- Better than before
- Same as before
- Not as well as before
- Much worse than before

1. **During the Covid-19 health crisis, have you been able to develop new skills (whether professional or not)?**

- Yes
- No

1. ***[ASK THIS QUESTION ONLY IF: “Yes” to Q5]*** **How satisfied are you with these new skills?**

- Very satisfied
- Satisfied
- Neither satisfied *nor* dissatisfied
- Dissatisfied
- Very dissatisfied

1. **During the Covid-19 health crisis, have you been able to give support (moral, material, etc.) to someone/other people?**

- Yes
- No

1. ***[ASK THIS QUESTION ONLY IF: “Yes” to Q7]*** **How satisfied are you with the support you have given?**

- Very satisfied
- Satisfied
- Neither satisfied *nor* dissatisfied
- Dissatisfied
- Very dissatisfied

1. **Since the start of the Covid-19 health crisis, have you been able to take advantage of the situation to do the following activities?**

- Take time for yourself, rest
- Do it yourself (DIY) projects, painting, tidying up
- Cooking and learning new recipes
- Getting closer to your family, discussing with each other, and getting together
- Playing sports / physical activity
- Reading, watching films and/or TV series
- Other
- No, I have not been able to do any of these activities **[exclusive choice]**

1. **Please indicate the extent to which you agree with each of the following statements :**

|  | **Strongly disagree** | **Disagree** | **Neutral** | **Agree** | **Strongly agree** |
| --- | --- | --- | --- | --- | --- |
| a. I tend to bounce back quickly after hard times | □ | □ | □ | □ | □ |
| b. I have a hard time making it through stressful events | □ | □ | □ | □ | □ |
| c. It does not take me long to recover from a stressful event | □ | □ | □ | □ | □ |
| d. It is hard for me to snap back when something bad happens | □ | □ | □ | □ | □ |
| e. I usually come through difficult times with little trouble | □ | □ | □ | □ | □ |
| f. I tend to take a long time to get over set-backs in my life | □ | □ | □ | □ | □ |

| B7 : Access to healthcare |
| --- |

We will now discuss your access to health services and prevention tools during the Covid-19 health crisis.

1. **Are you currently being followed for any of the following medical conditions?** *(multiple answers possible)*

- HIV
- STI
- Tuberculosis
- Hepatitis C virus (HCV)
- Hepatitis B virus (HBV)
- Cancer
- Diabetes
- Heart problems (hypertension, etc.)
- Other
- Not receiving care for a medical condition **[exclusive choice]**

1. **Have you needed to consult a healthcare professional for a chronic illness (other than suspected Covid-19) since the start of the Covid-19 epidemic?**

❏ Not applicable, because I do not have a chronic illness

❏ No, I have not needed to consult a healthcare professional

❏ Yes, I needed to consult a healthcare professional and had a consultation

❏ Yes, I needed to consult a healthcare professional but did not have/ have not had a consultation

**2.1** ***[ASK THIS QUESTION ONLY IF: “Yes, I needed to see a healthcare professional and had a consultation” to Q2]*** **How was the consultation conducted?** *(Multiple answers possible)*

- Via telemedicine (online consultation)
- A physical (face to face) appointment
- Other, specify: _________

1. ***[ASK THIS QUESTION ONLY IF: “Yes, I needed to consult a healthcare professional but did not have/have not had a consultation” to Q2]*** ***What* kind of care did it concern ?** *(Multiple answers possible)*

- Care provided at the hospital
- Care provided in a private practice, doctor’s office
- Home-based care
- Care provided by community-based organizations
- Other care, specify: ______

1. ***[ASK THIS QUESTION ONLY IF: “Yes, I needed to consult a healthcare professional but did not have/ have not had a consultation” to Q2]*** Why didn’t you /haven’t you consult/consulted a healthcare professional, even though you needed/need to? *(Multiple answers possible)*

- To protect myself from the risk of Covid-19 (avoid being contaminated)
- Because I had/have problems getting around (infrequent or unavailable transport)
- Because I didn’t/don’t have the means to protect myself from the risk of Covid-19 (mask, gloves)
- Because the home-based care services I was receiving have been reduced or stopped
- Because the healthcare professionals who followed/follow me reduced or stopped consultations
- Because the relevant services interrupted their activity
- Because I was refused treatment/care
- Because I did not ask for help/I decided not to have a medical consultation
- Other reason, specify:

1. **Have you needed to consult a healthcare professional because you suspected you had Covid-19?**

- Not applicable, as I do not think I had Covid-19
- No, I have not needed to see anyone
- Yes, I needed to see someone and had a consultation
- Yes, I needed/need to see someone but didn’t/ haven’t had a consultation

**5.2 *[ASK THIS QUESTION ONLY IF: “Yes, I needed/need to see someone but didn’t /haven’t had a consultation” to Q5]*** **Why didn’t/haven’t you consulted, even though you needed/need to?**

- To protect myself from the risk of Covid-19 (avoid being contaminated)
- Because I had/have problems getting around (infrequent or unavailable transport)
- Because I didn’t/don’t have the means to protect myself from the risk of Covid-19 (mask, gloves)
- Because the home-based care services I was receiving have been reduced or stopped
- Because the healthcare professionals who followed/follow me reduced or stopped consultations
- Because the relevant services interrupted their activity
- Because I was refused treatment/care
- Because I did not ask for help/I decided not to have a medical consultation
- Other reason, specify:

1. **Have you needed to go to a structure providing drug consumption-related risk reduction services and/or OST dispensation (opioid substitution treatment)?**

- Not applicable
- No, I have not needed these services
- Yes, I needed to go and was able to access the service
- Yes, I needed/need to go but didn’t/ haven’t accessed the service

1. ***ASK THIS QUESTION ONLY IF: “Yes, I needed/need to go but did not/ have not accessed the service” to Q6]*** **Why didn’t you access/haven’t you accessed the service even though you needed/need to?** *(Multiple answers possible)*

- To protect myself from the risk of Covid-19 (avoid being contaminated)
- Because I had/have problems getting around (infrequent or unavailable transport)
- Because I didn’t/don’t have the means to protect myself from the risk of Covid-19 (mask, gloves)
- Because the home-based care services I was receiving have been reduced or stopped
- Because the healthcare professionals who followed/follow me reduced or stopped consultations
- Because the relevant services interrupted their activity
- Fear of being stopped/questioned by the police
- Because I did not ask for help
- Other reason, specify:

1. **Have you had any serious or worrying health concerns (other than Covid-19) that could not be diagnosed and/or treated and/or operated on during the Covid-19 health crisis?**

- Yes
- No

1. **Since the start of the Covid-19 health crisis, how would you evaluate your access to the following tools and services?** *(Tick all boxes that apply)*

|  | **Easier** | **Same as before** | **More difficult** | **I have never used this tool/service** |
| --- | --- | --- | --- | --- |
| a. Condoms | □ | □ | □ | □ |
| b. Lubricants | □ | □ | □ | □ |
| c. PrEP | □ | □ | □ | □ |
| d. HIV testing | □ | □ | □ | □ |
| e. HIV treatment | □ | □ | □ | □ |
| f. STI testing | □ | □ | □ | □ |
| g. STI treatment | □ | □ | □ | □ |
| h. Hepatitis C virus (HCV) testing | □ | □ | □ | □ |
| i. Hepatitis C virus (HCV) treatment | □ | □ | □ | □ |
| j. Post-Exposure Prophylaxis (PEP) for HIV/ Emergency HIV treatment | □ | □ | □ | □ |
| k. Contraceptives | □ | □ | □ | □ |
| l. Needle and syringe exchange programme |  |  |  |  |
| m. Injection equipment | □ | □ | □ | □ |
| n. Opioid substitution Treatment (OST) | □ | □ | □ | □ |
| o. Access to Naloxone |  |  |  |  |

1. ***[ASK THIS QUESTION ONLY IF: “More difficult” to 9.a OR 9.b OR 9.c OR 9.e OR 9.f OR 9.h]*** **Why did you have difficulty accessing screening and other prevention tools?** *(Multiple answers possible)*

- Stockout
- Closure of services
- Inability to get to services (transport, hours of circulation, distance)
- Fear of exposing myself to Covid-19
- Lack of financial resources
- Fear of being stopped or questioned by the police
- Impossibility to have someone else look after my children
- Apathy (inability to react)
- Other, specify: ___________________

1. ***[ASK THIS QUESTION ONLY IF: “More difficult” to 9.d OR 9.g OR 9.i OR 9.k OR 9.j]*** **Why did you have difficulty accessing treatment?** *(Multiple answers possible)*

- Stockout
- Closure of services
- Inability to get to services (transport, hours of circulation, distance)
- Fear of exposing myself to Covid-19
- Lack of financial resources
- Fear of being stopped or questioned by the police
- Impossibility to have someone else look after my children
- Apathy (inability to react)
- Other, specify: ___________________

1. ***[ASK THIS QUESTION ONLY IF: “More difficult” to 9.l OR 9.m OR 9.n OR 9.o]* Why did you have difficulty accessing opioid substitution treatment (OST),** Needle and syringe exchange programme, **injection equipment or Naloxone?** *(Multiple answers possible)*

- Stockout
- Closure of services
- Inability to get to services (transport, hours of circulation, distance)
- Fear of exposing myself to Covid-19
- Lack of financial resources
- Fear of being stopped or questioned by the police
- Impossibility to have someone else look after my children
- Apathy (inability to react)
- Other, specify: ___________________

1. **Since the start of Covid-19 health crisis, have you had at least one telemedicine consultation (i.e., online medical consultation)?**

- Yes
- No

1. ***[ASK THIS QUESTION ONLY IF: “yes” to Q13]*** **If “yes”, were you satisfied with your telemedicine consultation(s)?**

- Very satisfied
- Satisfied
- Neither satisfied *nor* dissatisfied
- Dissatisfied
- Very dissatisfied

1. ***[ASK THIS QUESTION ONLY IF: “yes” to Q13]*** **Would you like to continue telemedicine consultations after the Covid-19 health crisis ends?**

- Yes, absolutely
- Yes, probably
- I don’t know
- No, probably not
- No, absolutely not

1. **Have you used or benefitted from any of the following since the start of the Covid-19 health crisis to stay informed and connected?** *(Multiple answers possible)*

- New Covid-19 specific websites
- Facebook groups
- Whatsapp groups
- Chatbot (chatting via a software package that can simulate conversation)
- Telephone/email contact with my medical care team
- Telephone/email contact with a community-based structure
- Online support groups (Zoom, Skype, etc.)
- Other, specify: _________________________
- No, none **[exclusive choice]**
- Not applicable **[exclusive choice]**

1. **Have you used or benefitted from any of the following since the start of the Covid-19 health crisis to adapt the way you seek care/treatment?** *(Multiple answers possible)*

- Consultation by telemedicine
- Consultation with a community-based worker/actor while respecting prevention measures
- Distribution of ARV by community-based workers/actors
- Dispensation of ARV for a greater number of months
- Earlier access to ARV at the hospital
- Earlier access to hepatitis C treatment
- Reduction in my HIV follow-up
- Reduction in my hepatitis C follow-up
- Other, specify: _________________________
- No, none **[exclusive choice]**
- Not applicable **[exclusive choice]**

1. **Have you used or benefitted from any of the following since the start of the Covid-19 health crisis to adapt the way you use drug consumption-related risk reduction services?** *(Multiple answers possible)*

- Distribution of opioid substitution therapy (OST) at home
- Distribution of opioid substitution therapy (OST) by community-based workers/actors
- Needle and syringe exchange programme implemented in outreach or off-site contexts (e.g. in the street, a squat, etc.)
- Needle and syringe exchange programme in an association’s premises while respecting prevention measures
- Easier access to Naloxone
- Take-home naloxone
- Other, specify: _________________________
- No, none **[exclusive choice]**
- Not applicable **[exclusive choice]**

1. **Have you used or benefitted from any of the following since the start of the Covid-19 health crisis to adapt the way you access HIV/ Hepatitis /STI prevention tools and devices?**

- Self-test kit delivery to my home (home self-testing)
- Access to condoms through outreach/off-site (for example, in the street) interventions
- Access to condoms in an association’s premises while respecting prevention measures
- Continuity of therapeutic trials
- Reduction of my PrEP follow-up
- Other, specify: _________________________
- No, none **[exclusive choice]**
- Not applicable **[exclusive choice]**

| B8 : People who use drugs (PWUD) |
| --- |

1. **During the past 12 months, have you used drugs?**

- Yes
- No **[End of module]**
- I do not wish to answer **[End of module]**

This section aims to assess the impact of the Covid-19 health crisis on your drug use and on your access to risk reduction tools. Remember that your answers are anonymous.

1. **During the past 12 months, have you injected drugs?**

- Yes
- No
- I do not wish to answer

1. **Before the Covid-19 health crisis, were you on opioid substitution treatment prescribed by a doctor?**

- Yes
- No

1. ***[ASK THIS QUESTION ONLY IF: “yes” to Q3]*** **If “yes”, this opioid substitution treatment was:**

- Methadone
- Buprenorphine
- Moscontin or Skenan
- Other, specify: _________________

1. ***[ASK THIS QUESTION ONLY IF: “yes” to Q3]*** **If “yes”, during the Covid-19 health crisis, did you personally have to interrupt your substitution treatment?**

- Yes
- No

1. ***[ASK THIS QUESTION ONLY IF: “yes” to Q3]*** **How would you evaluate your adherence to your opioid substitution treatment since the start of the Covid-19 health crisis?**

*(adherence to treatment is defined as taking the medication as directed by your doctor/prescription)*

- Worse than usual
- Slightly worse than usual
- The same
- Slightly better than usual
- Better than usual

1. **Old question deleted - keep this line to respect the numbering**
2. **Old question deleted - keep this line to respect the numbering**
3. **Old question deleted - keep this line to respect the numbering**

**9.2 Old question deleted - keep this line to respect the numbering**

1. ***[ASK THIS QUESTION ONLY IF: “yes” to Q2]*** **Compared with before the Covid-19 epidemic, has your access to sterile needles or syringes changed?**

- No, it has not changed
- Yes, I no longer have access
- Yes, I have less access
- Yes, I have more access

1. ***[ASK THIS QUESTION ONLY IF: “yes” to Q2]*** **Compared with before the Covid-19 epidemic, has your access to injection equipment changed?**

- No, it has not changed
- Yes, I no longer have access
- Yes, I have less access
- Yes, I have more access

**11.2** **Compared with before the Covid-19 epidemic, has your access to smoking/snorting equipment (crack pipes, snorting straw kits, etc.) changed?**

- No, it has not changed
- Yes, I no longer have access
- Yes, I have less access
- Yes, I have more access
- Not applicable

1. ***[ASK THIS QUESTION ONLY IF: “yes” to Q2]*** **When was the last time you injected drugs ?**

**___ /___/ _______ (DD/MM/YYYY)**

1. ***[ASK THIS QUESTION ONLY IF: “yes” to Q2]*** **Compared with before the Covid-19 epidemic, do you share your injection equipment…?**

- Much more often
- A little more often
- At the same frequency
- A little less often
- Much less often
- I do not share

1. **Compared with before the Covid-19 epidemic, do you share your smoking/snorting material (crack pipes, snorting straw kits, etc.)…?**

- Much more often
- A little more often
- At the same frequency
- A little less often
- Much less often
- I do not share
- Not applicable

1. **Compared with before the Covid-19 epidemic, how would you describe the frequency you consume the following substances?**

|  | **More often than before** | **Same as before** | **Less often than before** | **I have never taken this substance** |
| --- | --- | --- | --- | --- |
| a. Alcohol | □ | □ | □ | □ |
| b. Cannabis (pot, weed, marijuana, hash, etc.) | □ | □ | □ | □ |
| c. Opioids or analgesics (opium, heroin, morphine, methadone, fentanyl, skenan, etc.) | □ | □ | □ | □ |
| d. Benzodiazepines and sedatives | □ | □ | □ | □ |
| e. Poppers and other inhalants / solvents (nitrites, rush) | □ | □ | □ | □ |
| f. Synthetic drugs / New synthetic products (mephedrone, cathinones, 3-MMC, alpha-php, etc.) | □ | □ | □ | □ |
| g. Crystal Meth (rank, crystal, tweak, meth, ice, Tina, jib, etc.) | □ | □ | □ | □ |
| h. GHB/GBL (G, GH, juice, liquid ecstasy, liquid X, etc.) | □ | □ | □ | □ |
| i. Cocaine taken in powder form (coke, powder, snow, etc.) | □ | □ | □ | □ |
| j. Crack cocaine | □ | □ | □ | □ |
| k. Hallucinogens (LSD /acid, magic mushrooms) | □ | □ | □ | □ |
| l. Amphetamine and its derivatives (ecstasy, MDMA, speed, peaches, E, X, XTC, love pill, etc.) | □ | □ | □ | □ |
| m. Ketamine (Special K, K, Vitamin K, Ket, Kit Kat) | □ | □ | □ | □ |
| n. Sexual stimulants or drugs for erectile dysfunction (Viagra, Cialis, Kamagra, etc.) | □ | □ | □ | □ |

1. **How has the way you obtain your psychoactive substances changed since the start of the Covid-19 health crisis?** *(Multiple answers possible)*

- There has been no change **[exclusive choice]**
- I have not made any attempt to obtain psychoactive substances
- I have stopped getting psychoactive substances
- I changed my dealer/supplier
- I use the internet more than before to obtain psychoactive substances
- I buy my psychoactive substances more frequently on the dark web
- I buy larger quantities
- I buy smaller quantities
- I try to have psychoactive substances delivered to my home more frequently than before
- I try to reduce face-to-face pickups of illicit psychoactive substances
- Other, specify: _____________________

**[Note : delete the question below if you are using the module B7 access to healthcare]**

1. **Have you used any of the following since the start of the Covid-19 health crisis?** *(Multiple answers possible)*

- Telephone/email contact with my medical team
- Telephone/email contact with a community-based structure
- Telemedicine consultation
- Distribution of opioid substitution therapy at home
- Distribution of opioid substitution therapy by community-based workers/actors
- Needle and syringe exchange programme implemented in outreach or off-site contexts (e.g. in the street, a squat, etc.)
- Needle and syringe exchange programme in an association’s premises while respecting prevention measures
- Easier access to Naloxone
- Take-home naloxone
- Other, specify: _________________________
- No, none **[exclusive choice]**

1. **Compared to before the Covid-19 health crisis, have you ever experienced any of the following feelings in connection with your consumption of psychoactive substances?**

|  | **More often** | **Same as before** | **Less often** | **I have never had this feeling** |
| --- | --- | --- | --- | --- |
| a. Fear of being harassed or stopped/questioned by the police | □ | □ | □ | □ |
| b. Fear or experience of violent or hostile interactions (physical or verbal) by other people who use drugs | □ | □ | □ | □ |
| c. Fear or experience of discrimination by doctors | □ | □ | □ | □ |
| d. Fear or experience of discrimination by other healthcare professionals | □ | □ | □ | □ |
| e. Fear or experience of discrimination by your family or friends | □ | □ | □ | □ |

1. **Since the start of the Covid-19 health crisis, have you needed support from any of the following groups?**

|  | **Yes, and I have received the support I needed** | **Yes, but I (have) needed more support** | **Yes, but I have not received support** | **No, I have not needed support** | **Not applicable** |
| --- | --- | --- | --- | --- | --- |
| a. Friends (excluding current and former people who inject drugs) | □ | □ | □ | □ | □ |
| b. Family | □ | □ | □ | □ | □ |
| c. Peers (current or former people who inject drugs who are NOT involved in community-based organizations) | □ | □ | □ | □ | □ |
| d. Outreach workers involved in a community-based organization | □ | □ | □ | □ | □ |
| e. Doctors | □ | □ | □ | □ | □ |
| f. Other healthcare professionals | □ | □ | □ | □ | □ |

1. **Have you used drugs in a sexual context (including during chemsex)?**

- Yes, I did before the crisis, and I continue to do it with the same frequency (as before)
- Yes, I did before the crisis, and I’ve increased the frequency
- Yes, I did before the crisis, but I’ve lowered the frequency
- Yes, I did it before the crisis, but I’ve stopped doing it
- Yes, I began doing it only after the crisis started
- No, I’ve never done it

1. **This health crisis allowed me to reflect on my practices regarding my consumption of psychoactive substances:**

- Completely agree
- Agree
- Neither agree *nor* disagree
- Disagree
- Completely disagree
- Not applicable

#### **In your opinion, is the public health system’s response to the Covid-19 health crisis in your country (government, ministry of health, public hospitals, etc.) adapted to the reality of people who use drugs?**

- Yes, absolutely
- Yes, somewhat
- I don’t know
- No, not really
- No, not at all

#### **In your opinion, is the community response to the Covid-19 health crisis in your country adapted to the reality of people who use drugs?**

- Yes, absolutely
- Yes, somewhat
- I don’t know
- No, not really
- No, not at all

| B9 : People living with HCV |
| --- |

**0.1 Have you ever tested positive for hepatitis C virus (HCV)?**

- Yes
- No **[End of module]**
- I do not wish to answer **[End of module]**

This section aims to assess the impact of the Covid-19 health crisis on your HCV care and treatment pathway. Remember that your answers are anonymous.

**0.2 When was your last positive HCV test?**

- After the start of the Covid-19 health crisis
- In the past 12 months, but before the start of the Covid-19 health crisis
- Between 1 and 3 years ago
- More than 3 years ago
- I do not know

**0.3 Please indicate which of the following tests were conducted for your last positive HCV test :** *(Multiple answers possible)*

- Anti-HCV antibodies test (RDT or Elisa)
- Viral load test (PCR or GeneXpert or Genedrive)
- Genotyping
- Liver examination (Fibroscan, biopsy)
- All required tests
- I do not know

1. ***[ASK THIS QUESTION ONLY IF: “After the start of the Covid-19 health crisis” to Q0.2 ]*** **If you were tested positive for HCV at the start of the Covid-19 health crisis, have you been able to start your HCV treatment?**

- Yes
- No, because I was not able to finish all the necessary additional examinations before starting treatment
- No, because of a stockout
- Other
- Not applicable

1. **During the Covid-19 health crisis, have you interrupted your HCV treatment:**

- Yes
- No, because I have a stock of treatment
- No, I have spread out the remaining tablets over time
- No, I have been able to access treatment during the crisis
- Not applicable *(no current treatment, I had already finished my HCV treatment)*

1. ***[ASK THIS QUESTION ONLY IF: “yes” to Q2]*** **If “yes”, what were the reasons?** *(Multiple answers possible)*

- Lockdown (or isolation) with people who are not aware of my HCV status
- No longer have a prescription
- Stockout
- Closure of services
- Inability to get to services (transport, hours of circulation, distance)
- Fear of exposing myself to Covid-19
- Lack of financial resources
- Fear of being stopped or questioned by the police
- Impossibility to have someone else look after my children
- Apathy (inability to react)
- Other, specify: __________________

1. **Old question deleted - keep this line to respect the numbering of the questions.**
2. **Have you used or benefitted from any of the following since the start of the Covid-19 health crisis to stay informed and connected ?** *(Multiple answers possible)*

- Telephone/email contact with my medical team
- Telephone/email contact with a community-based structure
- Support groups online (Zoom, Skype, etc.)
- Other, specify : _________________
- No, none **[exclusive choice]**
- Not applicable **[exclusive choice]**

1. **Have you used or benefitted from any of the following since the start of the Covid-19 health crisis to adapt the way you seek care/treatment?** *(Multiple answers possible)*

- Consultation by telemedicine
- Consultation with a community-based worker/actor while respecting prevention measures
- Earlier access to treatment for hepatitis C
- Reducing my hepatitis C follow-up
- Other, specify: _________________________
- No, none **[exclusive choice]**
- Not applicable **[exclusive choice]**

1. **Have you used or benefitted from any of the following since the start of the Covid-19 health crisis to adapt the way you use risk reduction services?** *(Multiple answers possible)*

- Distribution of opioid substitution therapy at home
- Distribution of opioid substitution therapy by community-based workers/actors
- Needle and syringe exchange programme implemented in outreach or off-site contexts (e.g. in the street, a squat, etc.)
- Needle and syringe exchange programme in an association’s premises while respecting prevention measures
- Easier access to Naloxone
- Take-home naloxone
- Other, specify: _________________________
- No, none **[exclusive choice]**
- Not applicable **[exclusive choice]**

1. **Old question deleted - keep this line to respect the numbering**
2. **Old question deleted - keep this line to respect the numbering**

1. **Compared to before the Covid-19 health crisis, have you experienced any of the following feelings in relation to your HCV status?**

|  | **More often** | **Same as before** | **Less often** | **I have never had this feeling** |
| --- | --- | --- | --- | --- |
| a. Fear of being harassed or stopped/questioned by the police | □ | □ | □ | □ |
| c. Fear or experience of discrimination by doctors | □ | □ | □ | □ |
| d. Fear or experience of discrimination by other healthcare professionals | □ | □ | □ | □ |
| e. Fear or experience of discrimination by your family of friends | □ | □ | □ | □ |

1. **Since the start of the Covid-19 health crisis, have you needed support from any of the following groups?**

|  | **Yes, and I have received the support I needed** | **Yes, but I (have) needed more support** | **Yes, but I have not received support** | **No, I have not needed support** | **Not applicable** |
| --- | --- | --- | --- | --- | --- |
| a. Friends (excluding people currently and formerly living with HCV) | □ | □ | □ | □ | □ |
| b. Family | □ | □ | □ | □ | □ |
| c. Peers (people currently or formerly living with HCV who are NOT involved in community-based organizations) | □ | □ | □ | □ | □ |
| d. Local outreach workers involved in a community-based organization | □ | □ | □ | □ | □ |
| e. Doctors | □ | □ | □ | □ | □ |
| f. Other healthcare professionals | □ | □ | □ | □ | □ |

1. **In your opinion, is the public health system’s response to the Covid-19 health crisis in your country (government, ministry of health, public hospitals, etc.) adapted to the reality of people living with HCV?**

- Yes, absolutely
- Yes, somewhat
- I do not know
- No, not really
- No, not at all

1. **In your opinion, is the community response to the Covid-19 health crisis in your country adapted to the reality of people living with HCV?**

- Yes, absolutely
- Yes, somewhat
- I do not know
- No, not really
- No, not at all

| B10 : Pre-exposure prophylaxis (PrEP) |
| --- |

1. **Before the Covid-19 crisis, were you taking PrEP?**

- Yes, I was taking PrEP daily
- Yes, I was taking PrEP on demand (i.e., event-driven)
- No, I was not taking PrEP **[end of module]**

This section aims to assess the impact of the Covid-19 health crisis on your access to PrEP, your adherence to PrEP, and your related medical follow-up.

1. **If you are currently/were previously in lockdown, how are you using/were you using PrEP during the last lockdown?**

- Not applicable. I am not currently/was not previously in lockdown
- I take/was taking PrEP daily
- I take/was taking PrEP on demand (i.e., event-driven)
- I stopped taking PrEP

2.2  **If you are no longer in lockdown / you previously ended lockdown, how are you using/were you using PrEP after the end of lockdown?**

- Not applicable. I am not currently/was not previously in lockdown
- Not applicable. I have not yet ended lockdown
- I take PrEP daily
- I take PrEP on demand (i.e., event-driven)
- I stopped taking PrEP

1. ***[ASK THIS QUESTION ONLY IF: “I was taking PrEP daily” to Q1 AND “I take/was taking PrEP on demand (i.e., event-driven)”]*** **If you have switched from “daily PrEP” to “on-demand PrEP” during the Covid-19 health crisis, why did you do so?** *(Multiple answers possible)*

- I have sex less often
- I'm afraid of running out of medication during the Covid-19 health crisis to continue taking it daily
- Other, specify: ________________

1. ***[ASK THIS QUESTION ONLY IF: “I stopped taking PrEP” for Q2 OR “I stopped taking PrEP” for Q2.2*]** **If you stopped taking PrEP during or after lockdown, why did you do so?**  *(Multiple answers possible)*

- I no longer have/had at-risk sex at the moment
- I no longer have/had the desire to take PrEP
- I am/was in lockdown with people who are/were not aware that I take/took PrEP
- I no longer have/had a prescription
- Stockout
- Closure of services
- Inability to get to services (transport, hours of circulation , distance)
- Fear of exposing myself to Covid-19
- Lack of financial resources
- Fear of being stopped or questioned by the police
- Impossibility to have someone else look after my children
- Apathy (inability to react)
- Other, specify: __________________

1. **Before stopping PrEP, who did you contact to find out how (to stop) ?**  *(Multiple answers possible)*

- I did not contact anyone **[exclusive choice]**
- I contacted a doctor
- I contacted a healthcare provider
- I contacted an association
- I contacted peers
- I looked at websites/social networks on the subject
- Other

1. ***[ASK THIS QUESTION ONLY IF: “I stopped taking PrEP” for Q2 OR “I stopped taking PrEP” for Q2.2]*** **If you stopped taking PrEP, have you/ did you put into practice any other HIV risk reduction strategies?**  *(Multiple answers possible)*

- Condoms
- TasP (my HIV-positive partners are/were on treatment and have/had an undetectable viral load)
- Repeated HIV self-tests with partners
- Other, specify: __________________
- Not applicable **[exclusive choice]**

1. ***[ASK THIS QUESTION ONLY IF: “I stopped taking PrEP” for Q2 OR “I stopped taking PrEP” for Q2.2]*** **If you stopped/have stopped taking PrEP, do you plan to restart it after the Covid-19 health crisis ends?**

- Yes
- No
- I don’t know
- Not applicable

1. ***[ASK THIS QUESTION ONLY IF: “yes” to Q7]*** **If so, do you need information on how to restart PrEP?**

- Yes
- No

1. ***[ASK THIS QUESTION ONLY IF: “I was taking PrEP daily” for Q2 OR “I was taking PrEP on demand” for Q2]*** **If you are/were still taking PrEP during lockdown, did you have a stock of medicine built up before the start of the Covid-19 health crisis?**

- Yes
- No
- Not applicable

1. ***[ASK THIS QUESTION ONLY IF: “no” to Q9]*** ***If* you are/were still on PrEP and did not stock up on medicine before the start of the Covid-19 health crisis, do/did you have problems obtaining them?**

- Yes
- No
- Not applicable

1. ***[FILTRE SI : “Oui” à Q10]* Si oui, lesquelles ?**

***[ASK THIS QUESTION ONLY IF: “yes” to Q10]*** **If “yes”, what problems are you having?**

- Problems renewing the prescription
- Stockouts in some pharmacies
- Problems having the quarterly tests necessary for prescription renewal
- Problems getting to consultations (transport, hours of circulation, distance)
- Other, specify : __________________

1. **How would you evaluate your adherence to PrEP since the start of the Covid-19 health crisis?** *(adherence to treatment is defined as taking the medication as directed by your doctor/prescription)*

- Worse than usual
- Slightly worse than usual
- The same
- Slightly better than usual
- Better than usual
- Not applicable

1. **Since the start of the Covid-19 health crisis, with regard to your sex life…?**  *(Multiple answers possible)*

- I have not had sex
- I have made plans for sex for after the crisis
- I have reduced sexual encounters with new partners
- I have increased sexual encounters with new partners
- I have had more sex with the partner who lives with me
- I had more sexual activities/conversations/videos by messaging
- I have reduced the use of HIV prevention measures (condoms, PrEP, etc.) during sex
- None of the above **[exclusive choice]**
- Not applicable **[exclusive choice]**

1. **Have you used or benefitted from any of the following during the Covid-19 health crisis to stay informed and in contact?** *(Multiple answers possible)*

- New Covid-19 specific websites
- Facebook groups
- Whatsapp groups
- Chatbot (chatting via a software package that can simulate conversation)
- Telephone/email contact with my medical care team
- Telephone/email contact with a community-based structure
- Online support groups (Zoom, Skype, etc.)
- Other, specify: _________________________
- No, none **[exclusive choice]**
- Not applicable **[exclusive choice]**

1. **Have you used or benefitted from any of the following during the Covid-19 health crisis to adapt the way you seek care/treatment?** *(Multiple answers possible)*

- Consultation by telemedicine
- Consultation with a community-based worker/actor while respecting prevention measures
- Earlier access to PrEP
- Other, specify: _________________________
- None of the above **[exclusive choice]**
- Not applicable **[exclusive choice]**

1. **Have you used or benefitted from any of the following during the Covid-19 health crisis to adapt the way you access prevention tools and devices?** *(Multiple answers possible)*

- Self-test kit delivery to my home (home self-testing)
- Access to condoms through outreach interventions
- Access to condoms in an association’s premises while respecting prevention measures
- Continuity of therapeutic trials
- Reducing my PrEP follow-up
- Other, specify: _________________________
- None of the above **[exclusive choice]**
- Not applicable **[exclusive choice]**

1. **During the Covid-19 health crisis, have you needed to see a healthcare professional because you suspected that you had an STI?**

- Not applicable
- No, I have not needed to
- Yes, I needed to see someone and went for a consultation
- Yes, I needed/need to see someone but did not have/have not had a consultation

1. ***[ASK THIS QUESTION ONLY IF: “Yes, I needed to see someone and went for a consultation” for Q17]*** **How was the consultation conducted?** *(Multiple answers possible)*

- Via telemedicine
- A physical (face to face) appointment
- Other, specify: _________

1. ***[ASK THIS QUESTION ONLY IF: “Yes, I needed/need to see someone but did not have/have not had a consultation ” to Q17]*** **For what reasons did you not / have not consulted even though you need/needed to ?** *(Multiple answers possible)*

- To protect myself from the risk of Covid-19 (avoid being contaminated)
- Because I had/have problems getting around (infrequent or unavailable transport)
- Because I didn’t/don’t have the means to protect myself from the risk of Covid-19 (mask, gloves)
- Because the home-based care services I was receiving have been reduced or stopped
- Because the healthcare professionals who followed/follow me reduced or stopped consultations
- Because the relevant services interrupted their activity
- Because I was refused treatment/care
- Because I did not ask for help/I decided not to have a medical consultation
- Other reason, specify: _________

| B11 : People living with HIV |
| --- |

**0.1 Have you been diagnosed with HIV?**

- Yes
- No **[end of module]**
- I do not wish to answer  **[end of module]**

This section aims to assess the impact of the Covid-19 health crisis on your HIV care and treatment pathway. Remember that your answers are anonymous.

1. **What year were you diagnosed with HIV?**

__ __ __ __ **(YYYY)**

1. ***[ASK THIS QUESTION ONLY IF: “2020” to Q1]*** **Did you discover you were HIV positive after the start of the Covid-19 health crisis?**

- Yes
- No

2.2 **Have you ever (in your life) had a blood test that included a measure of your CD4 cell count?**

- Yes
- No
- I do not know

1. ***[ASK THIS QUESTION ONLY IF: “yes” to Q2.1]*** **When was the last time your CD4 cell count was measured?**

**month : ___________ (MM) year : ____________ (YYYY)**

3.2  **Have you ever (in your lifetime) had a blood test that included a measure of your viral load?**

- Yes
- No
- I do not know

1. ***[ASK THIS QUESTION ONLY IF: “yes” to Q3.1]*** **When was the last time your viral load was measured?**

**month : ___________ (MM) year : ____________ (YYYY)**

1. **The last time your CD4 count was measured, what was your CD4 cell count?**

- Less than 200/mm3
- Between 200 and 350/mm3
- More than 500/mm3
- I do not know
- Not applicable

1. **Your last viral load was :**

- Undetectable (less than 50 copies)
- Detectable
- I do not know
- Not applicable

1. **Are you on treatment for HIV?**

- Yes
- No

**7.2 *[ASK THIS QUESTION ONLY IF: “yes” to Q7]*** **What year did you start HIV treatment?**

**_______ (YYYY)**

1. ***[ASK THIS QUESTION ONLY IF: “yes” to Q7]*** **Have you interrupted your HIV treatment since the start of the Covid-19 health crisis?**

- Yes
- No

1. ***[ASK THIS QUESTION ONLY IF: “yes” to Q8]*** **Why did you interrupt treatment?**  *(Multiple answers possible)*

- Lockdown (or isolation) with people who are not aware of my HIV status
- No longer have a prescription
- Stockout
- Closure of services
- Inability to get to services (transport, hours of circulation, distance)
- Fear of exposing myself to Covid-19
- Lack of financial resources
- Fear of being stopped or questioned by the police
- Impossibility to have someone else look after my children
- Apathy (inability to react)
- Other, specify: __________________

1. **Old question has been deleted – keep this line to respect the numbering of questions**
2. ***[ASK THIS QUESTION ONLY IF: “yes” to Q7*]** **Since the start of the Covid-19 health crisis, have you had any problems getting antiretroviral drugs (ART)?**

- Yes
- No
- Not applicable

1. ***[ASK THIS QUESTION ONLY IF: “yes” to Q11]*** **If “yes”, what problems have you had? (many responses possible)**

- Problems renewing the prescription
- Stockouts in some pharmacies
- Problems getting to consultations (transport, hours of circulation, distance)
- Other, specify: __________________

1. ***[ASK THIS QUESTION ONLY IF: “yes” to Q7]*** **Have you reduced the amount of prescribed ART you take since the start of the Covid-19 health crisis?**

- Yes, on my own initiative
- Yes, in consulting with my doctor
- Yes, after discussing it with a peer educator
- No
- Not applicable

1. ***[ASK THIS QUESTION ONLY IF: “yes” to Q13]* If “yes”, how do you feel about this reduced intake?**

- It does not suit me at all
- It does not suit me
- It makes no difference to me
- It suits me
- It suits me perfectly

1. ***[ASK THIS QUESTION ONLY IF: “yes” to Q13] If* you have reduced your treatment, are you worried that you will no longer have an undetectable viral load?**

- Yes
- No
- I do not know

1. **Have you reduced the follow-up with your doctor / infectious disease specialist (number of appointments, telemedicine)?**

- Yes
- No
- Not applicable

1. ***[ASK THIS QUESTION ONLY IF: “yes” to Q16]*** **If “yes”, how do you feel about this reduced follow-up?**

- It does not suit me at all
- It does not suit me
- It makes no difference to me
- It suits me
- It suits me perfectly

1. ***[ASK THIS QUESTION ONLY IF: “yes” to Q7]*** **Have you had any difficulty taking your treatment because you are/ were in lockdown with people who are/were not aware of your HIV status?**

- Yes
- No
- Not applicable

1. ***[ASK THIS QUESTION ONLY IF: “yes” to Q7]*** **Since the start of the Covid-19 health crisis, would you say your adherence to ART is :**

*(adherence to treatment is defined as taking the medication as directed by your doctor/prescription)*

- Worse than before
- Slightly worse than before
- The same as before
- Slightly better than before
- Better than before

1. **Compared to before the Covid-19 health crisis, have you ever experienced any of the following feelings in relation to your HIV status?**

|  | **More often** | **Same as** **before** | **Less often** | **I have never had this feeling** |
| --- | --- | --- | --- | --- |
| a. Fear of being harassed or stopped/ questioned by the police | □ | □ | □ | □ |
| c. Fear or experience of discrimination by doctors | □ | □ | □ | □ |
| d. Fear or experience of discrimination by other healthcare professionals | □ | □ | □ | □ |
| e. Fear or experience of discrimination by your family of friends | □ | □ | □ | □ |

1. **Since the start of the Covid-19 health crisis, have you needed support from any of the following groups?**

|  | **Yes, and I received the support I needed** | **Yes, but I (have) needed more support** | **Yes, but I have not received support** | **No, I have not needed support from this group** | **Not applicable** |
| --- | --- | --- | --- | --- | --- |
| a. Friends (excluding people living with HIV) | □ | □ | □ | □ | □ |
| b. Family | □ | □ | □ | □ | □ |
| c. Peers (other people living with HIV who are NOT involved in community-based organizations) | □ | □ | □ | □ | □ |
| d. Local outreach workers involved in a community-based organization | □ | □ | □ | □ | □ |
| e. Doctors | □ | □ | □ | □ | □ |
| f. Other healthcare professionals | □ | □ | □ | □ | □ |

1. **Would you say that you have been sufficiently informed by the medical team taking care of you about the risk of Covid-19 infection in terms of your HIV infection and the medications you are taking?**

- Yes, absolutely
- Yes, somewhat
- I do not know
- No, not really
- No, not at all

1. **Has the Covid-19 health crisis forced you to reveal your HIV status to people who were not aware of it?**

- Yes
- No

1. **Old question deleted- keep this line to respect numbering of questions.**
2. **Have you used or benefitted from any of the following since the start of the Covid-19 health crisis to stay informed and connected ?** *(Multiple answers possible)*

- New Covid-19 specific websites
- Facebook groups
- Whatsapp groups
- Chatbot (chatting via a software package that can simulate conversation)
- Telephone/email contact with my medical care team
- Telephone/email contact with a community-based structure
- Online support groups (Zoom, Skype, etc.)
- Other, specify: _________________________
- No, none **[exclusive choice]**
- Not applicable **[exclusive choice]**

1. **Have you used or benefitted from any of the following since the start of the Covid-19 health crisis to adapt the way you seek care/treatment?** *(Multiple answers possible)*

- Consultation by telemedicine
- Consultation with a community-based worker/actor while respecting prevention measures
- Distribution of ARV by community-based workers/actors
- Delivery of ARV for a greater number of months
- Earlier access to ARV at the hospital
- Reducing my HIV follow-up
- Other, specify: _________________________
- No, none **[exclusive choice]**
- Not applicable **[exclusive choice]**

#### **In your opinion, is the public health system’s response to the Covid-19 health crisis in your country (government, ministry of health, public hospitals, etc.) adapted to the reality of people living with HIV?**

- Yes, absolutely
- Yes, somewhat
- I do not know
- No, not really
- No, not at all

#### **In your opinion, is the community response to the Covid-19 health crisis in your country adapted to the reality of people living with HIV?**

- Yes, absolutely
- Yes, somewhat
- I do not know
- No, not really
- No, not at all

| B12 : Sex workers |
| --- |

1. **Do you engage in sex work (i.e., you get something - for example: money, gifts, food, travel, housing accommodation, drugs, etc. - in exchange for sex*)?**

- Yes, I engaged in sex work before the Covid-19 health crisis and I continue to do so
- Yes, I began engaging in sex work after the start of the Covid-19 health crisis
- No, I engaged in sex work before the Covid-19 health crisis but I stopped
- No, I did not engage in sex work before the Covid-19 health crisis, and I do not engage in it now **[End of module]**

This section aims to assess the impact of the Covid-19 health crisis on your sex work activity, and the conditions for engaging in sex work. Remember that your answers are anonymous.

1. **Currently, is sex work your main activity (i.e., your main source of income)?**

- Yes, and it is my only activity
- Yes, but I have (an)other complementary activity(ies)
- No, I have a different main activity

1. **Compared with before the Covid-19 health crisis :**

- Sex work remains my main activity
- Sex work remains a complementary activity
- Today sex work has become a complementary activity for me
- Today sex work has become my main activity
- Not applicable

1. **Have you engaged in sex work in the following places?** *(Multiple answers possible for each line)*

|  | **Yes, before the Covid-19 crisis** | **Yes, since the start of the Covid-19 crisis** | **No, never [exclusive choice]** |
| --- | --- | --- | --- |
| Brothels/ massage parlours/ bars / other establishments |  |  |  |
| In the street or other public places |  |  |  |
| By appointment in my own place or location/in the customer's home /in a hotel |  |  |  |
| Online |  |  |  |

1. **Since the start of the Covid-19 health crisis, the income/goods you receive per week from sex work have...**

- Greatly increased
- Slightly increased
- Remained the same
- Slightly decreased
- Greatly decreased
- Not applicable

####

1. **Since the start of the Covid-19 health crisis, the number of clients you have per week for sex work has...**

- Greatly increased
- Slightly increased
- Remained the same
- Slightly decreased
- Greatly decreased
- Not applicable

1. **Compared with before the Covid-19 health crisis, how has the frequency with which you use the following risk reduction methods with your sex work clients changed?**

|  | **More frequent use** | **Same frequency as before the crisis** | **Less frequent use** | **I have never used this tool/ method** |
| --- | --- | --- | --- | --- |
| a. Condoms | □ | □ | □ | □ |
| b. Pre-exposure prophylaxis (PrEP)  This is a medical treatment taken by HIV-negative people which protects them from HIV infection | □ | □ | □ | □ |
| c. Post-exposure prophylaxis (PEP) or emergency treatment  This is a medical treatment taken by HIV-negative people after a high-risk exposure to infection | □ | □ | □ | □ |
| d. Antiretroviral therapy for treatment as prevention (TasP)  This is a medical treatment taken by HIV-positive people which protects their partners from HIV infection if taken correctly | □ | □ | □ | □ |
| e. Adaptation of sexual practices according to your serological status and that of your partners (also known as Seroadaptation) | □ | □ | □ | □ |
| f. Choosing partners according to their serological status (a Serosorting) | □ | □ | □ | □ |

**7.2 Compared with before the Covid-19 health crisis, how has the frequency with which you perform or engage in the following practices with your sex work clients changed?**

|  | **More frequent** | **Same frequency as before the crisis** | **Less**  **frequent** | **Not applicable** | **I do not wish to reply** |
| --- | --- | --- | --- | --- | --- |
| - 1. Use of drugs in a sexual context (including Chemsex) | □ | □ | □ | □ | □ |
| - 1. Injection drug use in a sexual context (called Slamming) | □ | □ | □ | □ | □ |

1. **Compared to before the Covid-19 health crisis, your risk of contracting HIV during sex with your clients is :**

- Much higher than before
- Slightly higher than before
- Same as before
- Slightly lower than before
- Much lower than before
- Not applicable

1. **Compared to before the Covid-19 health crisis, your risk of contracting HIV during sex with your other sexual partners (non-clients) is :**

- Much higher than before
- Slightly higher than before
- Same as before
- Slightly lower than before
- Much lower than before
- Not applicable

1. **Compared to before the Covid-19 health crisis, have you suffered the following types of violence from your clients?**

####

|  | **More often** | **Same as before** | **Less often** | **I have never suffered this kind of violence** | **I do not wish to answer** |
| --- | --- | --- | --- | --- | --- |
| a. Physical violence (strangulation, beatings, injuries, etc.) | □ | □ | □ | □ | □ |
| b. Verbal violence (name calling, insults, etc.) | □ | □ | □ | □ | □ |
| c. Psychological violence (harassment, threats, blackmail, etc.) | □ | □ | □ | □ | □ |
| d. Sexual violence (sexual relations or practices without consent, rape, condom removal without consent, etc.) | □ | □ | □ | □ | □ |

#### **In your opinion, is the public health system’s response to the Covid-19 health crisis in your country (government, ministry of health, public hospitals, etc.) adapted to the reality of sex workers?**

- Yes, absolutely
- Yes, somewhat
- I do not know
- No, not really
- No, not at all

#### **In your opinion, is the community response to the Covid-19 health crisis in your country adapted to the reality of sex workers?**

- Yes, absolutely
- Yes, somewhat
- I do not know
- No, not really
- No, not at all

#### **How have the measures implemented to combat the Covid-19 epidemic impacted the conditions for engaging in sex work? (many responses possible)**

- Sex work has become impossible
- Sex work has become more complicated
- Sex work is mostly done online now (webcam shows)
- The relationship with clients is more complicated (e.g., enforcing hygiene and Covid prevention measures)
- No impact **[exclusive choice]**

#### **Compared to before the Covid-19 health crisis, have you ever experienced any of the following in relation to sex work ?**

|  | **More often** | **Same as before** | **Less often** | **I have never had this feeling** |
| --- | --- | --- | --- | --- |
| a. Fear of being harassed or stopped/questioned by the police | □ | □ | □ | □ |
| b. Fear or experience of violent or hostile interactions (physical or verbal) by other sex workers | □ | □ | □ | □ |
| c. Fear or experience of discrimination by doctors | □ | □ | □ | □ |
| d. Fear or experience of discrimination by other healthcare professionals | □ | □ | □ | □ |
| e. Fear or experience of discrimination by your family of friends | □ | □ | □ | □ |

#### **Since the start of the Covid-19 health crisis, have you needed support from any of the following groups?**

|  | **Yes, and I received the support I needed** | **Yes, but I (have) needed more support** | **Yes, but I have not received support** | **No, I have not needed support from this group** | **Not applicable** |
| --- | --- | --- | --- | --- | --- |
| a. Friends excluding sex workers | □ | □ | □ | □ | □ |
| b. Family | □ | □ | □ | □ | □ |
| c. Peers (other sex workers who are NOT involved in community-based organizations) | □ | □ | □ | □ | □ |
| d. Local outreach workers involved in a community-based organization | □ | □ | □ | □ | □ |
| e. Doctors | □ | □ | □ | □ | □ |
| f. Other healthcare professionals | □ | □ | □ | □ | □ |

1. **Have you used or benefitted from any of the following since the start of the Covid-19 health crisis to stay informed and connected?** *(Multiple answers possible)*

- New Covid-19 specific websites
- Facebook groups
- Whatsapp groups
- Chatbot (chatting via a software package that can simulate conversation)
- Telephone/email contact with my medical care team
- Telephone/email contact with a community-based structure
- Online support groups (Zoom, Skype, etc.)
- Other, specify: _________________________
- No, none **[exclusive choice]**
- Not applicable **[exclusive choice]**

1. **Have you used or benefitted from any of the following since the start of the Covid-19 health crisis to adapt the way you seek care/treatment?** *(Multiple answers possible)*

- Consultation by telemedicine
- Consultation with a community-based worker/actor while respecting prevention measures
- Distribution of ARV by community-based workers/actors
- Dispensation of ARV for a greater number of months
- Earlier access to ARV at the hospital
- Earlier access to hepatitis C treatment
- Reduction in my HIV follow-up
- Reduction in my hepatitis C follow-up
- Other, specify: _________________________
- No, none **[exclusive choice]**
- Not applicable **[exclusive choice]**

**[Note :delete the question below if you use the module B7 access to healthcare]**

1. **Have you used or benefitted from any of the following since the start of the Covid-19 health crisis to adapt the way you use of risk reduction services?** *(Multiple answers possible)*

- Distribution of opioid substitution therapy (OST) at home
- Distribution of opioid substitution therapy (OST) by community-based workers/actors
- Needle and syringe exchange programme implemented in outreach or off-site contexts (e.g. in the street, a squat, etc.)
- Needle and syringe exchange programme in an association’s premises while respecting prevention measures
- Easier access to Naloxone
- Take-home naloxone
- Other, specify: _________________________
- No, none [exclusive choice]
- Not applicable [exclusive choice]

1. **Have you used or benefitted from any of the following since the start of the Covid-19 health crisis to adapt the way you access prevention tools and devices? *(Multiple answers possible)***

- Self-test kit delivery to my home (home self-testing)
- Access to condoms through outreach/off-site (for example, in the street) interventions
- Access to condoms in an association’s premises while respecting prevention measures
- Continuity of therapeutic trials
- Reduction of my PrEP follow-up
- Other, specify: _________________________
- No, none **[exclusive choice]**
- Not applicable **[exclusive choice]**
